# Desmin Correlated with Cx43 May Facilitate Intercellular Electrical Coupling during Chronic Heart Failure

**DOI:** 10.1155/2021/6621132

**Published:** 2021-07-03

**Authors:** Junxian Cao, Qianping Gao, Hongyan Chen, Can Wang, Qiuju Zhang, Zhipeng Wang, Yuanshi Li

**Affiliations:** ^1^Department of Cardiology, The First Affiliated Hospital of Harbin Medical University, Harbin, Heilongjiang 150001, China; ^2^Department of Cardiology, Heilongjiang Province Land Reclamation Headquarters General Hospital, Harbin, Heilongjiang 150088, China; ^3^Department of Statistics, Harbin Medical University School of Public Health, Harbin, Heilongjiang 150086, China; ^4^Xijing Hospital, Xi'an, Shaanxi 710032, China

## Abstract

Desmin is one of five major intermediate filament proteins in cardiomyocytes. Desmin contributes to the maintenance of healthy muscle. The desmin content in cardiomyocytes directly affects the long-term prognosis of patients with heart failure, and lack of desmin leads to myocyte contractile dysfunction. However, the mechanism is elusive. In this study, we measured desmin expression using western blotting and qPCR in the failed hearts of human patients and rats. Our results showed that desmin content was reduced at the protein level in failed hearts and isolated cardiomyocytes. The association of desmin and the gap junction proteins connexin 43 (Cx43) and zonula occludens-1 (ZO-1) was also investigated. Immunoprecipitation assay showed that desmin was associated with Cx43 in cardiomyocytes. To compare the electrical integration of skeletal myoblasts in cocultures with cardiac myocytes, familial amyloid polyneuropathy (FAP) activation rate was found in 33% desmin overexpressing skeletal myoblasts. Desmin not only affected Cx43 and ZO-1 expression but also facilitated the complex of Cx43 and ZO-1 in skeletal myoblasts, which enhanced cell-to-cell electrical coupling of skeletal myoblasts with cardiac myocytes. Desmin has potential as a novel therapeutic target for heart failure. Preservation of desmin may attenuate heart failure.

## 1. Introduction

Chronic heart failure (HF) has high morbidity and mortality that is strongly determined by the high propensity of remodeled hearts to have fatal ventricular tachyarrhythmias [1]. The cardiac remodeling process is governed by structural and electrical changes in impulse conductions such as a change in electrical coupling due to abnormal expression of intermediate filament (IF) and connexin 43 (Cx43) constituted gap junctions [2, 3].

Desmin is one of five major IF proteins in cardiomyocytes and forms a cytoskeletal network that connects actin filaments with Z-disk and regulates sarcomere architecture. Through its connection to the sarcomere, desmin connects the contractile apparatus to the cardiomyocyte nucleus and mitochondria. Changes in desmin may affect myocardial contraction as well as mitochondria function [4, 5]. Cardiomyocytes lacking the IF protein desmin have numerous mitochondrial structural abnormalities such as disturbed localization, extensive proliferation, sub-sarcolemmal aggregation, swelling, and matrix disorganization that are involved in cardiomyocyte hypertrophy and chamber dilation, resulting in cardiomyopathy and heart failure [6–10]. The mechanism by which desmin IFs contribute to the maintenance of healthy muscle and the mechanism by which lack of desmin leads to cardiomyopathy remain unclear. Desmin content in cardiomyocytes is observed to directly affect the long-term prognosis of HF patients. Desmin expression was variable in failing heart tissue. Heling et al. showed that, in failing hearts with hypertrophy and dilated cardiomyopathy, desmin was upregulated in both mRNA and protein levels [8] in presence of hypertrophy or dilated cardiomyopathy. Sharov et al. also showed that in dog failing hearts desmin showed increased expression in areas of extensive fibrosis [9]. Pawlak et al. found different expressions of desmin levels by immunohistology in samples of human HF. However, low desmin expression in cardiomyocytes, measured by immunohistochemical assays, is associated with unfavorable clinical courses [10].

In the heart, stability and integrity of cell-to-cell interactions and membrane junctional proteins are crucial for many biological processes including the cardiac remodeling process during HF [11]. Gap junctions form the intercellular pathways for propagation of the precisely orchestrated patterns of current flow that decides the regular rhythm of the healthy heart [12]. Gap junctions are ensembles of cell-cell channels that are formed by the connexin family. Cx43 is a member of the connexin family that is expressed throughout the heart tissue. The working (contractile) myocytes of the ventricle are extensively interconnected by clusters of Cx43-containing gap junctions [13, 14]. Cx43 affects cardiac electrical properties [15]. Abnormal Cx43 expression is often associated with abnormal conduction and arrhythmias. Pronounced reduction of ventricular Cx43 at the protein level is typical of the hearts of patients with end-stage congestive HF [16].

Adherence junctions are a prerequisite for gap junction assembly in cultured cardiomyocytes and mediate intercellular adhesion and via anchoring actin filaments contribute to propagation of the contraction between cardiomyocytes by linking to the actin cytoskeleton and the IF system at desmin [17–19]. Gap junctions mediate intercellular communication, playing an essential role in electrical and metabolic coupling by regulating the movement of ions and small molecules through hydrophilic channels between cells. Expression of a dominant negative N-cadherin in rat cardiomyocytes disrupts gap junction organization at the cell surface. Furthermore, gap junctions are lost in N-cadherin-null embryonic cardiomyocytes and reassembled upon reintroduction of cadherin [20]. N-cadherin dominants are shown to target Cx43 to adherence junctions through interacting with microtubule plus-end tracking proteins [21]. Recent evidence shows that knockdown of N-cadherin reduces trafficking of an N-cadherin/Cx43 multiprotein complex to the cell surface of NIH3T3 cells [22]. These results suggest that N-cadherin may be involved in multiple steps in the development of functionally mature gap junction plaques at the plasma membrane. In addition, zonula occludens-1 (ZO-1) has been demonstrated to regulate the organization of gap junctions and adherence junctions at ICDs [23].

In this study, we hypothesized that desmin facilitated development of gap junctions and assembly of Cx43 channels that could be beneficial in failing heart. We determined desmin expression in failed hearts and investigated the correlation with the gap junction proteins Cx43 and ZO-1.

## 2. Materials and Methods

### 2.1. Human Samples

The study was approved by the Institutional Review Board at Xijing Hospital, Xi'an. Written informed consent was obtained from participating individuals of heart donors. All research was conducted in compliance with Good Clinical Practice standards. Failing human hearts were procured at the time of orthotropic heart transplantation at the Xijing Hospital, Xi'an, following informed consent from all participants. Nonfailing (NF) hearts were obtained at the time of organ donation from cadaveric donors. In all cases, transmural myocardial samples were dissected from the mid left ventricle (LV) free wall below the papillary muscle. LV tissues for western blots and quantitative PCR (qPCR) were flash-frozen in liquid nitrogen.

### 2.2. Animals

Male Wistar rats weighing 180–220 g were housed in a temperature- and humidity-controlled room using a 12 h light/dark cycle. The protocol was approved by the Institutional Animal Care and Use Committee of the Research Institute at Harbin Medical University.

### 2.3. Animal Heart Failure Model Surgery Protocol

An aortocaval fistula (ACF) rat model was established as previously described [24]. Animals were subjected to aorta vena cava fistula. A suprasternal incision was made, exposing the abdominal aorta. In the animals in the HF group, a disposable needle (outer diameter 0.6 mm, Braun Beckinson, Germany) was inserted into the exposed abdominal aorta and advanced into the vena cava to create the fistula. The needle was withdrawn and the aortic puncture site was sealed with a drop of cyanoacrylate glue (Krazy Glue, Border, Willowdale, Canada). Animals in the sham NF group underwent a similar procedure without puncture. In the streptomycin (SM) group, rats were administered streptomycin intraperitoneally as streptomycin sulfate (S-6501, Sigma Chemical Co., St. Louis, MO) injection 3 times daily for 8 days (300 mg per kg per day) prior to sacrifice.

### 2.4. Echocardiography

Echocardiographic studies were performed before operations and before sacrifice using an ultrasound machine (SONOS 7500, Philips) fitted with a 12 MHz transducer. Septum, posterior wall thickness (PW) left ventricular diameter at end diastole (LVDD), left ventricular diameter at end systole (LVESD), and left ventricular fractional shortening (LVFS) were measured in three consecutive cardiac cycles, and the average value of each parameter was recorded. We used LVEF ≤50% to determine HF.

### 2.5. Primary Cultures of Neonate Rat Cardiomyocytes, Skeletal Myoblasts, and Transfection

Primary rat skeletal myoblasts were isolated from the hind leg muscles of adult male Wistar rats. Muscle slices were digested in 0.25% pancreatin and 1% trypsin for 1 hour with occasional agitation. Isolated cells were collected by filtering through 70 *μ*m nylon cell strainers (Falcon). Counterselection against fibroblasts was by 2 rounds of differential adhesion on collagen-coated tissue culture flasks (40 min at 37°C for each adhesion step). Primary myoblasts were cultured in a CO_2_ incubator at 37°C in DMEM with glutamax-I (Invitrogen) supplemented with 20% fetal calf serum (FCS) and further purified by sorting using paramagnetic beads (Dynal Biotech) coated with antibody H36 against myoblast-specific *α*-7 integrin [22].

Cardiac myocytes were isolated from neonatal rats. Freshly excised ventricles were dissociated in trypsin-EDTA (Invitrogen), and dispersed cells were suspended in a 4 : 1 mixture of DMEM and M199 medium (Invitrogen) supplemented with 15% horse serum and 5% FCS. The cell suspension was preplated to separate fibroblasts from myocytes as described for skeletal myoblasts above. The myocytes remaining in suspension were cultured in a CO_2_ incubator at 37°C.

Small interfering RNA (siRNA) targeting desmin (AAGCAGGAGAUGAUGGAAU) was synthesized by Shanghai GenePharma Co. Ltd. Cells were transfected using Lipofectamine 2000 (Invitrogen) with an siRNA targeting rat desmin. Cells were plated in 500 *μ*L growth medium without antibiotics one day before transfection and were 25%–45% confluent at the time of transfection. Transfected cells were cultured in DMEM without FCS for 8 hours.

For the transfection of myoblasts with a desmin-EGFP plasmid, myoblasts were cultured and transfected using Lipofectamine 2000 (Invitrogen) with an MLV-desmin-EGFP plasmid or empty MLV plasmid (both produced by Shanghai GenePharma Co. Ltd). Transduced cells were sorted using a preparative FACS machine (Becton Dickinson FACS DIVA cell sorter) to produce a cell population.

### 2.6. Immunoprecipitation

Immunoprecipitation was performed with 50 *μ*g protein from lysed cells as described previously [25], diluted in RIPA buffer. After overnight incubation at 4°C on a rotating device, immune complexes were precipitated at 4°C for 4 h on a rotating device with protein A/G magnetic beads (Pierce). Immunoprecipitated complexes were washed three times with RIPA buffer before extraction in Laemmli buffer at room temperature for western blot analysis.

### 2.7. LV Myocyte Isolation

At 21 weeks after HF surgery, viable LV myocytes were isolated as previously described [12]. Briefly, hearts were mounted on a Langendorff apparatus followed by retrograde perfusion. The LV was separated from the digested heart, and myocytes were mechanically dispersed and filtered. Isolated myocytes were resuspended and plated on laminin-coated cell-perfusion chambers for a 1-hour incubation. Plated myocytes were cultured in serum-free MEM with 0.1% bovine serum albumin and 100 U/mL penicillin-streptomycin.

### 2.8. Immunoblots

LV midwall tissue lysates were prepared as described [26]. Proteins (15 *µ*g) were separated by SDS-PAGE and transferred to PVDF membranes. Immunoblotting was performed as described [25] with antibodies against desmin (1 : 2000 Abcam, ab15200, Boston, USA), N-cadherin (1 : 5,000, Santa Cruz, sc-271386), Zo-1 (1 : 5,000, Santa Cruz, sc-33725), or GAPDH (1 : 4000; Zhongshan, TA08, Beijing, China). Relative band densities were analyzed using GelEval (v1.22 Frog Dance Software).

### 2.9. qPCR

Total RNA isolation, cDNA synthesis, PCR, and real-time PCR were performed as described previously [25]. The amount of mRNA was estimated and normalized to an endogenous reference (GAPDH) relative to a calibrator. Primers used were desmin-F1 5′-CATCGCGGCTAAGAACATTT-3′, desmin-R1 5′-GCCTCATCAGGGAATCGTTA-3′, GAPDH-sense 5′-GAAGGTGAAGGTCGGAGTCA-3′ and GAPDH-antisense 5′-TGGAAGATGGTGATGGGATT-3′.

### 2.10. Immunofluorescence Microscopy

Immunofluorescence was performed as described [27]. Following antigen retrieval and blocking with 10% goat serum, slides were incubated overnight at 4°C with a rabbit polyclonal anti-Cx43 (sc-6560, 1 : 250, Santa Cruz). For secondary labeling, goat anti-rabbit AlexaFluor 488 (1 : 500, Invitrogen, Carlsbad, CA) conjugated IgG was used and sections were mounted and counterstained using Vectashield with DAPI (Vector Laboratories, Burlingame, CA).

### 2.11. Electrophysiological Measurements in Cell Cocultures

As described previously [28], to compare the electrical integration of skeletal myoblasts (desmin/EGFP transduced and nontransduced) in cocultures with cardiac myocytes, cell culture dishes incorporating a group of 30 *μ*m diameter 60 embedded unipolar electrodes with interelectrode distances of 100 *μ*m (Multielectrode Array, MCS GmbH, Reutlingen, Germany) were used. Two groups of cell cultures were investigated: (1) cardiac myocytes cocultured at a ratio of 4 : 1 with desmin-transduced skeletal myoblasts and (2) cardiac myocytes cocultured at the same ratio with nontransduced skeletal myoblasts. To establish cocultures, cardiac myocytes were seeded at 1 million cells per dish in multielectrode array dishes and cultured.

At day 2 after seeding of cardiac myocytes, desmin-transduced skeletal myoblasts or nontransduced skeletal myoblasts were added at 0.25 million cells per culture dish. At day 3, an extracellular stimulatory current was applied in 10 evenly spaced pulses (80 *μ*A, 5 ms) at 10 s time intervals. To register familial amyloid polyneuropathy (FAP), electrograms (potential against time) were recorded for 10 s by the recording system. The FAP activation rate (frequency of FAP firing) was tested using a spike sorter of the MC-Rack data analyzer (Microcal Software, Northampton, MA, USA). Data were analyzed as the average of that obtained from the 60 electrodes.

### 2.12. Statistical Analysis

Data were expressed as mean ± standard deviation (SD). Statistical analyses were performed using SPSS.11.0. Unpaired Student's *t*-tests or one-way ANOVA, followed by Bonferroni's post hoc test, were used to measure differences between groups, and *p* < 0.05 was considered statistically significant.

## 3. Results

### 3.1. Desmin Expression in Donor LV Tissue

**Figure 1:**
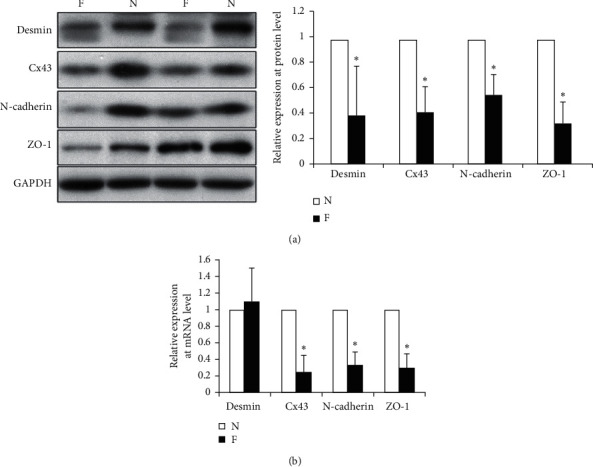
Desmin, ZO-1, N-cadherin, and Cx43 expression in human hearts. (a) Lysates from donor hearts that were normal (N) or failing (F) were isolated and used for western blots with anti-desmin, anti-Cx43, anti-N-cadherin, or anti-ZO-1. GAPDH antibody was the loading control. (b) qPCR was used to test for mRNA for desmin, Cx43, N-cadherin, and ZO-1. ^*∗*^*p* < 0.05 vs nonfailing heart.

We isolated and lysed cells in tissue from donors of 2 HF hearts and 2 non-HF hearts. Western blots showed lower desmin content in HF tissues than in non-HF tissues. Cx43, N-cadherin, and ZO-1 expression was downregulated in HF tissues (*p* < 0.05) (Figure 1(a)). However, qPCR results showed desmin mRNA was not altered between HF and non-HF tissues. RNA for Cx43, N-cadherin, and ZO-1 was lower in HF tissues (*p* < 0.05) (Figure 1(b)).

### 3.2. Desmin Expression in the LV of Rats with Heart Failure

We next compared desmin expression at the protein and mRNA levels in animal models. After 21 weeks of aortic sealing, rats showed echocardiographic signs of LV hypertrophy, including increases in wall thickness (both posterior and septal) and posterior wall thickness, increases in LV dimensions, and fractional shortening (Table 1).

**Figure 2:**
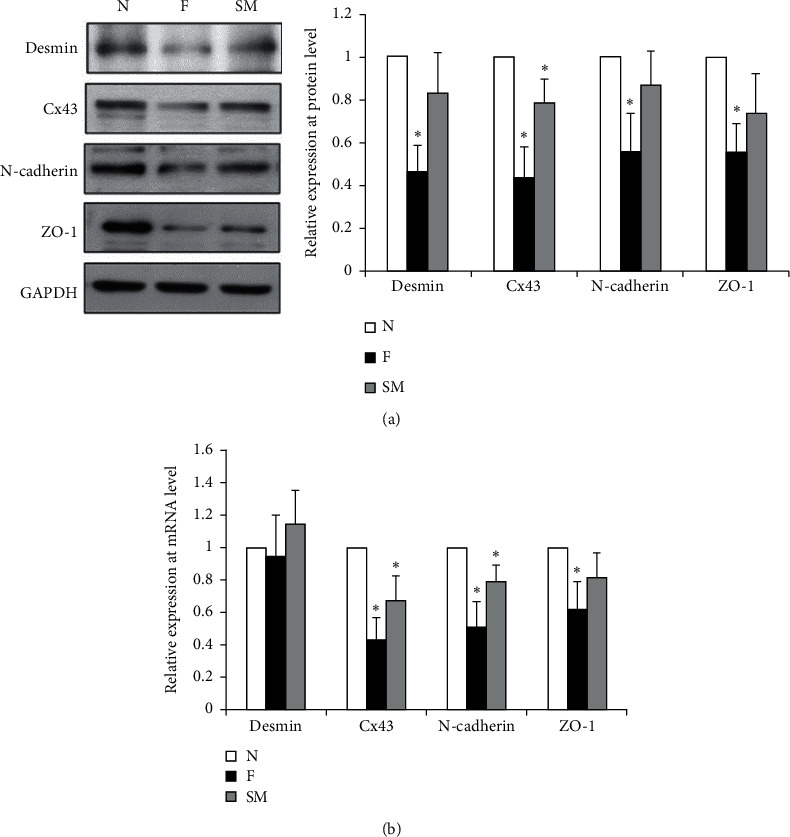
Desmin, ZO-1, N-cadherin, and Cx43 expression in rat hearts. Lysates were prepared from nonfailing (N) and failing (F) hearts and rats treated with streptomycin (SM). (a) Western blots used antibodies against desmin, Cx43, N-cadherin, and ZO-1. (b) qPCR was used to test for mRNA for desmin, Cx43, N-cadherin, and ZO-1. ^*∗*^*p* < 0.05 vs nonfailing heart.

Desmin content was determined by western blots. As shown in Figure 2, the desmin content was lower in the HF group than the sham group (N). Desmin was higher in the SM group than in the HF group (*p* < 0.05) (Figure 2(a)). By qPCR, desmin RNA levels were the same in all groups. However, RNA for Cx43, N-cadherin, and ZO-1 was lower in HF tissues and higher in the SM group (*p* < 0.05) (Figure 2(b)).

### 3.3. Desmin Affected Cx43 and ZO-1 in Cardiomyocyte and Skeletal Myoblasts

**Figure 3:**
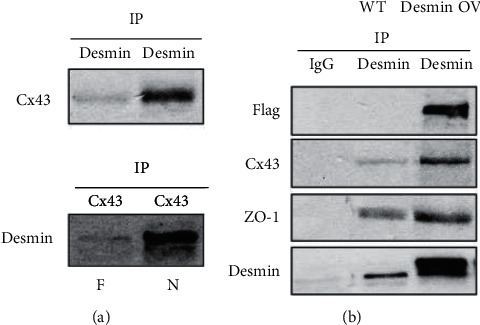
Desmin and Cx43 form a scaffolding complex in skeletal myoblasts. (a) Desmin and Cx43 coimmunoprecipitate in isolated skeletal myoblasts. Protein lysates from isolated cardiac myocytes were coimmunoprecipitated (IP) with anti-desmin or anti-Cx43. Desmin and Cx43 IP samples were used in immunoblots with either anti-desmin or anti-Cx43. (b) Lysates of cultured wild-type (WT) skeletal myoblasts or desmin-overexpressing (OV) skeletal myoblasts were used to determine if desmin formed a complex with Cx43 and ZO-1. Desmin IP samples were used for immunoblots with anti-Cx43 and ZO-1. IgG was used as the negative control. N, normal hearts; F, failed hearts.

Association of desmin and Cx43 was detected in cardiomyocytes by immunoprecipitation assays (Figure 3(a)). In skeletal myoblasts, complexes of Cx43 and ZO-1 with desmin were observed when desmin was overexpressed in skeletal myoblasts (Figure 3(b)).

**Figure 4:**
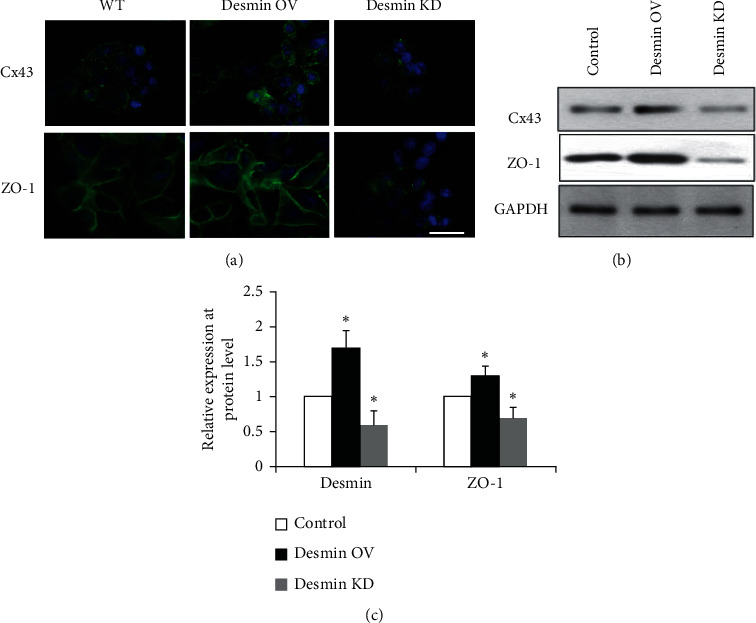
Cx43 and ZO-1 expression was affected by desmin in skeletal myoblasts. (a) Immunofluorescence was used to test Cx43 and ZO-1 expression in wild-type (WT), desmin-overexpressing (OV), or desmin knockdown (KD) skeletal myoblasts. Green: Cx43, ZO-1; blue: DAPI. Scale bars: 20 *µ*m. (b) Western blots were used to test Cx43 and ZO-1 expression in wild-type, desmin OV, or desmin KD skeletal myoblasts. (c) Quantification of results of (b). ^*∗*^*p* < 0.05 vs control.

We measured Cx43 and ZO-1 proteins in desmin-overexpressing or desmin-knockdown cells. Immunofluorescence and western blots showed that Cx43 and ZO-1 were upregulated in desmin-overexpressing cells (*p* < 0.05) (Figure 4). Both Cx43 and ZO-1 were downregulated in desmin-repressed cells.

### 3.4. Desmin Overexpression in Skeletal Myoblasts Improves Electrical Coupling in Cocultures

**Figure 5:**
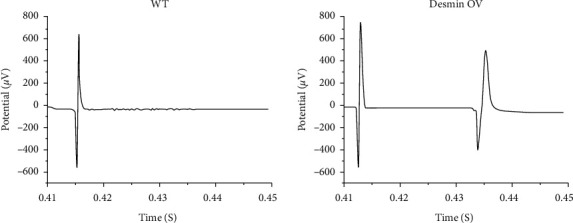
Multielectrode array recording of cocultures of primary skeletal myoblasts with cardiac myocytes. Electrograms show the last stimulatory pulse in a series of 10 and the first resulting FAP in cocultures of cardiac myocytes with nontransduced skeletal myoblasts (WT) or desmin-overexpressing skeletal myoblasts (desmin OV). Ten stimulatory current pulses were applied with a frequency of 1 Hz.

Cocultures of skeletal myoblasts and cardiac myocytes were established to mimic *in vivo* transplantation of skeletal myoblasts into the host myocardium. Spontaneous FAPs were observed in 33% (4 of 12) of cocultures of desmin-transduced skeletal myoblasts (OV) with cardiac myocytes and 10% (1 of 10) of cocultures of nontransduced wild type skeletal myoblasts (WT) with cardiac myocytes (Figure 5). In nontransduced myoblasts, FAP activation had a sporadic pattern. Stimulation with 10 pulses of current applied for 10 s was sufficient to obtain FAPs in 100% (12 of 12) of cocultures of desmin-transduced skeletal myoblasts with cardiac myocytes and 40% (4 of 10) of cocultures of nontransduced skeletal myoblasts with cardiac myocytes. In nontransduced wild-type skeletal myoblasts, the pattern of FAP activation was sporadic. No FAP activation was observed in 10 individual cultures of nontransduced skeletal myoblasts and 10 individual cultures of transduced skeletal myoblasts.

## 4. Discussion

As a major IF in the functional interaction of mitochondria and the cytoskeleton, desmin is crucial in physical and pathological processes that lead to cardiomyopathy and heart failure [5]. In this study, desmin expression did not change at the mRNA level in both normal and failed human or rat heart tissue. However, protein expression was reduced in failed heart tissue compared to nonfailing hearts, indicating that desmin was altered at the posttranscriptional level, which needs further investigation. In this rat model, decreased desmin level correlated with echocardiographic signs of LV hypertrophy, such as increased wall thickness, decreased LV dimensions, and increased fractional shortening, which indicated heart failure. Similarly, Panagopoulou et al. found that TNF-*α*-induced cleavage of desmin is an important mechanism for its loss from ICDs. In transgenic mice that express a desmin mutant (D263E) harboring a substitution of the aspartic acid (D) 263 with a glutamic acid (E), which renders desmin resistant to caspase-mediated cleavage, Cx43 displayed a normal distribution at ICDs. This finding suggests that desmin might be cleaved by overexpression of TNF-*α*, which leads to development of a dilated cardiomyopathy that recapitulates the classical transition to failure with progressive LV dysfunction and remodeling, cardiac myocyte hypertrophy, interstitial fibrosis, and progressive myocyte loss during the HF process [29].

This study showed that reduced desmin content in myocytes resulted in downregulation of Cx43, N-cadherin, and ZO-1, consistent with the finding that the expression of Cx43 is always associated with desmin-positive myocytes throughout the embryonic stage of mice [30]. Moreover, desmin facilitates complexes of Cx43 and ZO-1. Reducing desmin led to fewer junction proteins, resulting in instability of cell-to-cell interactions and integrity of junctional membrane proteins. This is a major alteration in the cardiac remodeling process in HF and related fatal ventricular tachyarrhythmias.

To repair the damaged myocardium in HF patients, cell transplantation is a promising method [31]. With varying degrees of differentiation and fusion into the host tissue, implanted cells can engraft into the host myocardium. On the one hand, transplanted myoblasts integrate and differentiate into multinucleated myotubes. On the other hand, these myoblasts do not transdifferentiate to cardiac myocytes or couple with the host cardiac myocytes, leading to a lack of electrical coupling of the implanted cells with the host myocytes. As a result, grafted skeletal myoblasts do not incorporate enough into the beating cardiac muscle [32]. Tolmachov et al. showed that cocultures of cardiac myocytes with Cx43-transduced skeletal myoblasts had enhanced cell-to-cell electrical coupling due to overexpression of Cx43 in skeletal myoblasts [28]. In our study, desmin overexpression resulted in improved electrical coupling between transduced skeletal myoblasts and cardiac myocytes *in vitro.* This result suggested that overexpression of desmin in skeletal myoblasts is a possible step for engineering electrocompetent cardiac grafts for treatment of HF.

## 5. Conclusion

Desmin was associated with Cx43 in cardiomyocytes. Desmin not only affected Cx43 and ZO-1 expression but also facilitated the complex of Cx43 and ZO-1 in skeletal myoblasts, which enhanced cell-to-cell electrical coupling of skeletal myoblasts with cardiac myocytes. Desmin has potential as a novel therapeutic target for heart failure, which needs to be further investigated in more animal models and patient biopsies.

## Figures and Tables

**Table 1:** Echocardiographic measures in rats after surgery.

	Septum, mm	PW, mm	LVEDD, mm	LVESD, mm	LVFS, %
Sham	13.83 ± 1.5	14.65 ± 1.3	60.69 ± 4.9	33.18 ± 4.7	38.97 ± 7.0
HF	22.06 ± 4.1 ^*∗*^	20.22 ± 2.9 ^*∗*^	70.12 ± 5.6 ^*∗*^	39.51 ± 5.8 ^*∗*^	47.16 ± 7.2 ^*∗*^
SM	17.06 ± 5.2	18.34 ± 3.3 ^*∗*^	65.02 ± 3.2	35.56 ± 4.1	41.16 ± 3.2

HF: heart failing group; SM: streptomycin-treated group; PW: posterior wall thickness during diastole; LVEDD, LV diameter at end diastole; LVESD, LV diameter at end systole; LVFS, LV fractional shortening. ^*∗*^*p* < 0.05 vs sham.

## Data Availability

The datasets used and/or analyzed during the current study are available from the corresponding author upon reasonable request.
